# Neighborhood Governance and Happiness during the COVID-19 Pandemic: An Empirical Analysis of Wuhan’s Lockdown

**DOI:** 10.3390/bs13060512

**Published:** 2023-06-19

**Authors:** Hanbei Cheng, Anli Jiang

**Affiliations:** 1 School of Public Policy and Management, Tsinghua University, Beijing 100084, China; 2 Center for Governance Studies, Beijing Normal University at Zhuhai, Zhuhai 519087, China; anlijiang@bnu.edu.cn

**Keywords:** ‘people-centered’ neighborhood governance, self-rated happiness, social inequalities, ordinal logistic models, Wuhan

## Abstract

The outbreak of COVID-19 posed a challenge to global governance, residents’ happiness, and economic systems around the world. Since the crux of previous research centers on the reactions of both local and national governments, studies on how governance arrangement at the neighborhood level influences people’s happiness during the crisis response remain insufficient. This paper aims to explore the relationship between neighborhood governance and residents’ happiness based on first-hand data collected during Wuhan’s first lockdown. This study highlights the significance of neighborhood governance in crisis response, which includes providing diverse public services, ensuring access to life’s necessities, and offering prompt medical treatment. All of these factors are essential for maintaining overall satisfaction with governance and contributing to the happiness of individuals within the community. However, active governance actions do not always lead to favorable results. For example, increased group participation may lead to social conflicts among those involved, ultimately diminishing one’s happiness. Furthermore, the COVID-19 pandemic has acted as a risk ‘amplifier’, exposing and exacerbating pre-existing *hukou*-based social inequalities in the governance process. The impact of the pandemic on citizen happiness is the cumulative effect of both the immediate social crisis brought on by the pandemic and long-standing structural inequalities. To improve people’s happiness and establish inclusive policies, this paper advocates for a ‘people-centered’ urban governance that enhances public satisfaction and addresses the needs and priorities of migrant populations.

## 1. Introduction

The world today is characterized as transitioning toward a ‘risk society’ [1], and the outbreak of COVID-19 in January 2020 significantly impacted global socioeconomic development and public health. As the epidemic spread rapidly from specific cities to local communities across countries, it posed challenges to urban governance systems.

Urban governance, a crucial mechanism for restructuring the urban world [2], has been extensively studied. However, most previous research has considered urban governance as a component of existing strategic packages in tandem with government initiatives promoting local wellbeing [3,4,5,6]. Despite this, theories and empirical evidence concerning the associations between urban governance and happiness are inadequate due to the broad demands and expectations of the community, particularly regarding governance satisfaction at the neighborhood scale [7,8]. Recently, urban scholars and environmental psychologists have become increasingly interested in the tangible/intangible benefits of urban governance for citizens’ happiness promotion and have unveiled its underlying social processes [3,4,9,10,11]. This sustained focus on promoting people’s happiness via urban governance worldwide highlights the issue’s prominence within public policy [6].

During the COVID-19 pandemic, scholars have explored the response methods of specific cities and countries. Most studies have focused on the implementation of governance by top-down political power at the national or local governments and the horizontal cooperation modes among various participating actors, such as local governments, community grassroots organizations, and other social entities in controlling pandemics [12,13,14,15]. However, limited attention has been given to the impact of specific governance arrangements, such as service provision, medical support, and public participation, implemented in a given city during the early stages of an outbreak response on residents’ happiness. This issue is crucial, as it not only exposes the inadequacies of the existing emergency governance systems in cities but also identifies the most urgent governance needs of residents during crises. Addressing this issue might guide the optimization of future governance systems.

Focusing on Wuhan, the initial epicenter of COVID-19, the city implemented various measures to contain the disease during the lockdown, including mobility control, the establishment of Fangcang (shelter hospitals), and citizen–state collaborative governance [14,16,17]. These efforts, collectively known as the ‘Wuhan experience’, served as a model for other places [18]. While we have acknowledged the short-term implications of COVID-19, it is crucial to investigate its long-term implications, particularly on urban governance due to the public health emergency events characterized by randomness and uncertainty [14]. In this context, analyzing case studies of Chinese cities like Wuhan can provide valuable insights. By studying epidemic prevention and control strategies and exploring generalizable urban governance responses, we can derive immediate and practical significance that contributes to the health and safety of cities worldwide.

Therefore, this paper aims to examine the impacts of neighborhood governance during crises, with a focus on people’s happiness. To achieve this, we conducted a questionnaire survey during the COVID-19 lockdown in Wuhan. Our study explores the determinants of neighborhood governance, which are categorized into two dimensions: objective measures and perceived satisfaction. We examine how these factors affect people’s self-rated happiness and also shed light on the role of residents’ satisfaction with crisis response in shaping their overall happiness. Additionally, our study uncovers whether there is social inequality in the governance process, particularly related to the *hukou* system, a unique household registration system that originally institutionalized China’s rural–urban dual society system to historically restrict internal migration and divide access to resources opportunities [19,20]. To explore this, we classified individuals’ migration status into two categories based on the *hukou* type according to their registered addresses: those holding Wuhan *hukou* (referred to as locals) and those without (referred to as migrants). By doing so, we hope to initiate a theoretical dialogue between urban governance and public health and to increase our understanding of happiness-oriented urban governance in urban China.

## 2. Literature Review

In recent decades, researchers worldwide have recognized individuals’ subjective wellbeing or self-rated happiness as the most meaningful and trustable indicator of a person’s life quality [21,22]. The word is also used in the more specific meaning of “a subjective enjoyment of life”, often used interchangeably with “well-being” and denotes both individual and social welfare [23]. While traditional factors related to individuals (such as sociodemographic attributes) have been acknowledged as influencing happiness [24,25], an increasing body of research has emphasized the critical role of place-based factors, including urban governance, in shaping happiness [26,27].

Governance is implemented in multiple superimposed geographical scales [28]. Existing research on governance and wellbeing (including people’s happiness) often examines multiscale geographical entities. For example, studies at the macro-national and regional scales have mostly focused on how governance influences citizens’ wellbeing from various facets of human life, including political stability, democracy (i.e., voice and accountability), control of corruption, and legislative transparency [26,29,30,31,32]. Several studies have focused on the wellbeing effects of urban governance at the meso-scale (e.g., the city) by examining various aspects, including quality of governance (QoG), fiscal decentralization, civic initiatives, and self-governance [26,33,34]. It is worth noting that governance issues such as community decentralization, group engagement, informal neighborhood control, participatory health intervention, and primary healthcare services have been discussed thoroughly on the neighborhood micro-scale [3,4,5,10]. Despite the wealth of evidence drawn from Western cities, it appears to be insufficient in conveying the multifaceted nature of China’s urban governance amidst ongoing urban transformation and the perpetuation of top-down political authority.

Researchers have reviewed the varying associations between urban governance and wellbeing outcomes. While a majority of these studies suggest that good governance contributes to citizens’ happiness, some have yielded even contradicted findings [10,35,36,37]. The scholar has suggested the ‘place mechanisms’ in urban governance, which posits that wellbeing outcomes (e.g., happiness) are related to the unique development history of cities and localized governance systems [11]. This makes it challenging to generalize findings from studies conducted in developed countries to cases like China, which is characterized using a context-specific governance structure combining state/local government guidance and community autonomy. Consequently, there is a need to expand case studies to encompass global cities at various stages of urbanization and governance contexts to address this gap in the literature. This current study aims to tackle this issue.

As stated by the Chinese Ministry of Civil Affairs (2000) [38], a part of the mandate of neighborhood governance is to mobilize physical and social resources to respond to the risk of uncertain public crises and maintain community stability. It has been argued that China’s neighborhood governance is at crossroads with the current situation in many other countries [5,39,40]. Therefore, it is pertinent to ask whether neighborhood governance in Chinese cities benefits its citizens, particularly during risky situations like the ongoing pandemic. Does the implementation of active governance measures necessarily promote residents’ happiness, and what role does public satisfaction play in this? Furthermore, are there social inequalities based on the *hukou* system in neighborhood governance? These questions remain under-researched.

First, numerous studies in China have focused on neighborhood governance and extensively discussed its governance models and initiatives [39,41,42]. For example, drawing on the concepts of state entrepreneurialism and entrepreneurial society, this study proposed four types of governance modes related to homeowner associations (HOAs, in Chinese *Yewei Hui*): compliant HOAs, contentious HOAs, self-managed HOAs, and self-governed HOAs [42]. Based on evidence from 32 Nanjing communities, a study identified and described four governance modes: collective consumption, service privatization, civic provision, and state-sponsored [41]. It is undeniable that their seminal work has made a significant contribution to our theoretical and practical understanding of urban governance in China. Nevertheless, it is crucial to acknowledge that the evidence presented in these studies mainly pertains to non-crisis situations, which may limit its applicability in times of crisis. Additionally, while the concept of ‘happiness’ is frequently emphasized as a strategic goal of people’s aspiration for a better life in these studies, its intrinsic relationships have not been fully explored. Considering that ‘happiness’ is a crucial social implication of urban governance; it is imperative that it be integrated into the research framework to thoroughly evaluate the effectiveness of governance strategies.

Second, some studies have explored the social implications of urban governance, particularly at the neighborhood scale [3,4,5,39]; however, their associations are, to some extent, exaggerated due to the neglect of social factors, such as the role of governance satisfaction, which is commonly viewed as a proxy to measure governance effectiveness [16,26,43]. For instance, one study observed that high-quality service delivery contributes to the happiness of those who are impoverished or lived in disadvantaged, overcrowded, and precarity spaces or neighborhoods [5]. The findings of a national survey identified that the number of amenities and organizations in neighborhoods is positively correlated with the mental health of Chinese seniors [4]. Similarly, conflicts between residents and local governments, particularly when it comes to neighborhood planning and shared property issues, were significantly associated with increased depression symptoms [3]. Only a small number of recent studies have investigated governance initiatives and relevant social processes in the context of crisis response. It is demonstrated that the services offered by the committees (in Chinese, Juwei Hui) and volunteer groups can greatly mitigate the negative impact of the COVID-19 epidemic on residents’ mental health, identifying social cohesion as one of the contributing social mechanisms [16]. However, the existing evidence is limited, with little information available on other social factors in-depth, including public satisfaction with governance initiatives in crisis response.

Finally, existing research has predominantly examined the general population’s experiences of neighborhood governance, neglecting the potential differences in happiness effects between locals and migrants who hold diverse socio-political positions within the governance system. The COVID-19 pandemic has highlighted the exacerbation of social inequalities in Western countries and China, which is particularly relevant in the context of pre-existing governance structures [44,45,46].

Social inequality is a critical issue in neighborhood governance, particularly for vulnerable groups like migrants. In China, migrants often cluster in disadvantaged areas, such as urban villages (i.e., *Chengzhongcun* in China) or affordable housing, due to limited access to local housing markets. These areas are often characterized by poor governance environments that increase migrants’ exposure to public health crises. Moreover, China’s *hukou* system impedes migrants’ access to services and resources that are typically available to local residents [20]. Furthermore, migrants frequently encounter inadequate social and financial support in their destination cities, exacerbating their vulnerability and hindering their recovery when facing adverse situations that undermine their social, economic, and material resilience. Given these challenges, it is essential to investigate the impact of social inequality based on the *hukou* system in neighborhood governance and establish a scientific foundation for constructing inclusive communities.

**Figure 1:**
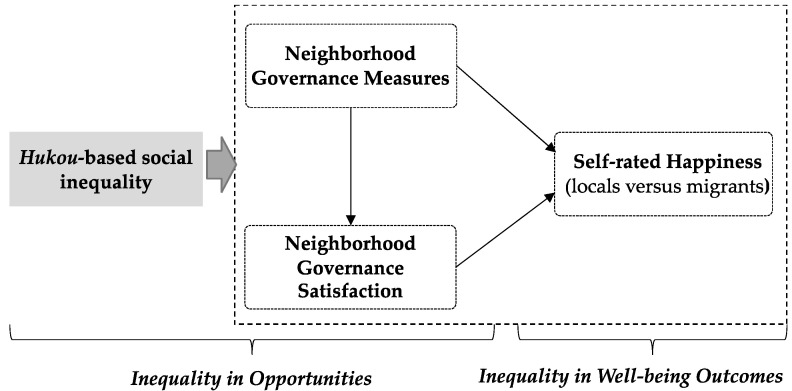
The research framework.

The objective of this study is to contribute to the existing body of knowledge by empirically examining the associations between neighborhood governance and self-rated happiness. In line with China’s new-type urbanization strategy, the fundamental goal of urban development is to enhance people’s happiness (more information can be found at https://www.ndrc.gov.cn/wsdwhfz/202303/t20230306_1350702.html, accessed on 2 January 2023), which becomes particularly important during crises. Prioritizing citizens’ happiness not only contributes to maintaining political stability but also improves the capacity to effectively respond to risks. Neighborhood governance plays a crucial role in strengthening social capital, enhancing collective efficacy, and thereby improving social resilience and ensuring people’s happiness [47]. Guided by the principle of ‘people-centered’ urbanization, we conceptually decompose neighborhood governance into objective (measures) and perceptual (satisfaction) dimensions and highlight the social inequalities perpetuated by China’s *hukou* system. The hypotheses were proposed as follows, and the corresponding research work is presented in Figure 1.

**H1.**  *During the pandemic, residents’ self-rated happiness is directly linked to neighborhood governance (including both measures and satisfaction).*

**H2.**  *Active governance measures do not necessarily lead to increased happiness. Instead, satisfaction is the key factor and serves as an essential social mechanism to explain the connection between governance measures and self-happiness.*

**H3.**  *The impact of neighborhood governance on self-happiness may differ between locals and migrants in Wuhan, highlighting the hukou-based social inequality in urban governance in China.*

## 3. Research Design

### 3.1. Data

**Figure 2:**
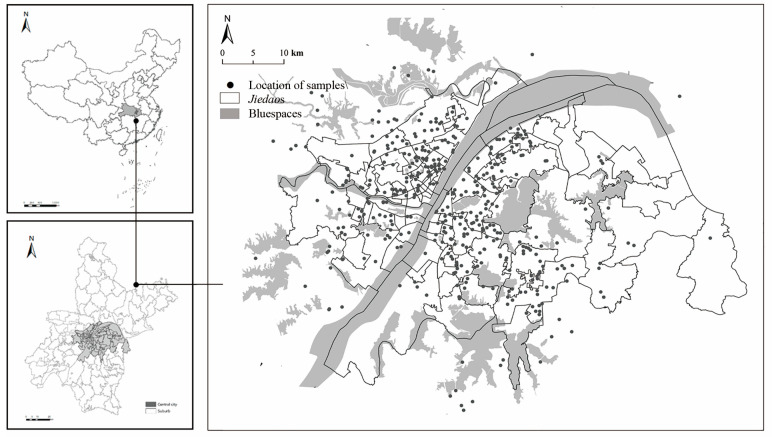
The discrete distribution of respondents.

We used data from the Epidemic Control and Residents’ Life of Quality Survey (EC&QoL-Wuhan), which was conducted from 18 to 24 February 2020, during the city’s first lockdown. The survey used a network-based snowball sampling technique via WeChat forwarding. During the data collection process, several students and faculty members from universities in Wuhan were identified as ‘seeds’. These seeds then invited their acquaintances to respond to an online questionnaire. The survey was open to eligible respondents who were 17 years and older and currently residing in Wuhan. To increase the geographical coverage of the survey, the seeds were requested to refer acquaintances from diverse age groups and both genders. Prior to the survey, an online pre-survey meeting was conducted to validate the questionnaire and establish ethical guidelines for these ‘seeds’. At the onset of the questionnaire, we emphasized its only use for academic research purposes and explicitly stated that all study outcomes would be presented solely as collective statistics, ensuring complete anonymity. We obtained informed oral consent from all survey participants. We gathered information regarding respondents’ socioeconomic status, neighborhood initiatives, attitudes towards multilevel governance actions, and various wellbeing outcomes. A total of 831 respondents’ data were collected. After excluding cases with IP addresses originating outside of Wuhan, and responses with incomplete information, we finally captured 790 valid samples, yielding a 95.1% response rate. The spatial distributions of respondents with different *hukou* types are shown in Figure 2.

Wuhan has promoted the ‘Wuhan Experience of Joint Governance’ in recent years, advocating for the cooperation of its government with citizens, committees, central and provincial governments, market entities, and other stakeholders at the National Conference on Municipal Social Governance Modernization, which was held in 2019. Moreover, the spread of the COVID-19 virus and the ensuing city-wide lockdown created new challenges for Wuhan’s governance system. These features make Wuhan a typical case.

### 3.2. Measurement

Due to the limited availability of data, we utilized a self-rating scale to assess each participant’s happiness during the epidemic by asking the question: “How do you feel about your overall happiness during the epidemic?” (1–5: very unhappy to very happy). This measurement has been widely employed and well validated in related studies [48,49]. It is particularly prevalent in China’s national surveys, including the Chinese General Social Survey (CGSS, 2013) and the China Social Governance Survey (CSGS, 2022). Notably, to ensure the validity of our results, we conducted a robustness check by utilizing self-rated mental health as an alternative outcome variable (as described in Section 3.3 and Table A1 in Appendix A).

Neighborhood governance is our interest, which consists of two aspects: objective measures and perceptual satisfaction (Figure 1). Notably, ‘satisfaction’ has been identified as an essential yardstick for ‘good governance’ [7,50], making it a crucial aspect to focus on. As a significant contribution to knowledge, this research emphasizes both the ‘hard’ and ‘soft’ aspects of neighborhood governance by intuitively integrating them into a research framework adapted to crises. The detailed variable settings are outlined below.

We utilized five variables to assess neighborhood governance measures, as informed by the research of [3,10,16,43]. These variables included public service delivery, mobility control, group involvement, life’s necessities provision, and prompt medical treatment. On the one side, we evaluated residents’ access to public services, encompassing regular disinfection, body temperature monitoring, food delivery, and information dissemination about epidemics. We combined these four service items to measure community public service delivery. Higher values indicated better community public service provision. We examined community entrance/exit controls as a measure of community mobility control, using a dichotomous variable. Communities with controlled entrance/exit were coded as 1 (yes), while those without were coded as 0 (no). We measured group involvement by calculating the total number of group memberships involved in neighborhood governance during the epidemic. Participants were asked to list up to five groups, such as workplace organizations, committees (in Chinese, Juwei Hui), property companies, volunteers, and others. Higher values signified greater levels of participation. Similarly, we surveyed residents about material shortages experienced during the epidemic, covering nine items: lack of meat, rice, vegetables, masks, protective clothing, hand sanitizers, disinfectants, coronavirus pharmaceuticals, and related chronic medications. These items were summed as the material deprivation index (range 0–9), measuring the provision of life’s necessities. Higher values indicated a weaker community in ensuring residents’ material completeness during the pandemic, revealing poor governance. Additionally, prompt medical treatment was assessed as an evaluation of timely medical treatment in response to COVID-19 outbreaks in neighborhoods. This variable was coded on a scale ranging from 1 (very bad) to 5 (very good). On the other side, we employed governance satisfaction as a perception variable via the question: “How satisfied are you with neighborhood governance during the epidemic?” The Likert scale response varied from 1 (highly dissatisfied) to 5 (highly satisfied).

In addition, we controlled a range of sociodemographic variables that are well documented to influence happiness, including age, gender, education, self-reported physical health, monthly household income, living arrangement, and *hukou* type. Age was grouped into two categories: those 36 years of age and below (coded as 0) and those over 36 (coded as 1). Gender was quantified as a dichotomous variable with 1 denoting female; education was grouped into ‘under junior secondary schools’ (coded as 1), ‘senior secondary schools and college’ (coded as 2), and ‘undergraduate and above’ (coded as 3). Self-reported physical health was scored on a scale ranging from 1 (very unhealthy) to 5 (very healthy). Monthly household income was classified into three categories to reflect the respondent’s economic situation (i.e., low: ≤5000 yuan; medium: 5000~20,000 yuan; high: >20,000 yuan). Additionally, adults living with their parents were expected to report lower happiness due to prolonged nursing and caregiving during the pandemic. Therefore, the respondent’s living arrangement (living with parents; 1 = yes, and 0 = no) was also controlled. Lastly, we categorized the *hukou* type into locals and migrants based on their registered addresses. We also control the residents’ risk exposure to COVID-19 using a dichotomous variable based on the question: Are there any suspected or confirmed cases in your community? Yes (coded as 1, representing high risk) or No (coded as 0, representing low risk). Detailed information is presented in Table 1.

### 3.3. Analytic Approach

Previous studies revealed that a person’s wellbeing (including happiness) was commonly influenced by two distinct tiers: individual-level factors and contextual-level factors (i.e., neighborhood environment) [51]. Individuals are embedded in their neighborhoods. Thus, multilevel models were preferred above single-level models when evaluating the link between neighborhood environment and individuals’ happiness, which aided in reducing the model’s estimation bias [52]. However, due to mobility control and social distancing restrictions during the pandemic, we had to collect data via an online survey, resulting in samples with highly discrete geographic distribution (Figure 2). This distribution did not correspond to the hierarchical data structure of individuals and neighborhoods in multilevel models [52]. Therefore, it is more reasonable to employ a single-level model in this study to enable more accurate empirical inferences.

Our detailed analysis unfolds in three steps. Step 1: We applied ordered logit regression models (OLM) in this study, considering that our dependent variable, self-rated happiness, was rated on a 1–5 ordinal scale. Step 2: We implemented a three-stepwise mediation analysis [53] to explore the social mechanism of governance satisfaction. First, we regressed residents’ self-rated happiness on neighborhood governance measures and covariates (Model 2). Second, we regressed ‘satisfaction’ on governance measures and covariates (Model 4). Third, we regressed self-rated happiness on governance measures, satisfaction, and covariates (Model 3). Step 3: We introduced interaction terms between governance variables and *hukou* type to examine whether the effects of governance measures and satisfaction on happiness differed between locals and migrants (Models 5 and 6). The average variance inflation factor (*VIF*) value for all independent variables in Model 3 was 2.09. However, education exhibits a high *VIF* value of 6.9 that exceeds a certain threshold (commonly considered to be around 5 or 10) [54], suggesting that the variable is highly correlated with other variables in the model with multicollinearity. To guarantee accurate and reliable results, the education variable was excluded from all models.

To ensure the reliability and validity of our findings, we conducted a robustness check by altering the outcome variable. We employed self-rated mental health due to its strong correlation with general happiness, which is widely recognized as a positive emotional aspect of mental wellbeing [24]. To perform this check, we re-estimated a range of models, as presented in Table A1 in Appendix A. Notably, self-rated happiness and self-rated mental health in our research exhibited a significant correlation coefficient of 0.649 (*p* < 0.001), affirming the feasibility of this robustness testing approach. All the analyses were performed using STATA 15.0 with the ‘ologit’.

## 4. Results

### 4.1. Descriptive Analysis

Table 1 displays the descriptive statistics of the final 790 samples. The average score for self-rated happiness was 3.37 (SD ± 1.23), indicating that Wuhan residents experienced general happiness during the pandemic. In terms of neighborhood governance, the average number of public services received by residents was 2.26 (SD ± 1.38). Slightly fewer than half (46.2%) of the participants reported receiving three or more types of services. During the lockdown period, a significant proportion (70.76%) of the participants reported that their community implemented regular disinfection and sanitation services. This was closely followed by temperature monitoring, reported by 61.39% of the respondents. In contrast, the provision of food delivery services by the community was reported by the lowest percentage of participants (42.03%). Moreover, 87.34% of the respondents’ communities enforced mobility restrictions. The average level of group involvement was 1.72 (SD ± 0.84). Specifically, committees (in Chinese, Juwei Hui) and property companies were the most commonly reported entities involved in governance, being mentioned by 57.34% and 58.23% of the sample, respectively. Additionally, 32.15% of respondents reported the involvement of volunteers, while less than 10% indicated governance by other entities or workplaces. The average score for daily living shortages reported by residents was 3.56 (SD ± 2.27, ranging from 0 to 9), signifying that the community effectively provided and maintained essential materials for its residents. Additionally, the average prompt medical treatment score was 3.07 (SD ± 1.08), reflecting a general level of responsiveness. Lastly, concerning public satisfaction with governance, the average score was 2.94 (SD ± 1.26), indicating an overall moderate level of satisfaction.

### 4.2. Neighborhood Governance and Self-Rated Happiness

Table 2 presents the regression results, suggesting strong correlations between neighborhood governance and residents’ self-rated happiness. Specifically, the coefficients of living essentials provision, as evaluated by the material shortage index and self-rated happiness was negative, indicating that residents were less likely to report higher happiness scores if their neighborhood provided insufficient daily materials during the pandemic (OR = 0.895, *p* = 0.000). Additionally, the coefficient of public service diversity was positive, implying that residents who had access to a greater variety of community services were more likely to report increased happiness (OR = 1.149, *p* = 0.017). Furthermore, we observed that prompt medical treatment in neighborhoods was associated with higher residents’ happiness scores (OR = 2.007, *p* = 0.000), making it the strongest predictor among governance parameters.

Contrary to our expectations, Model 2 reveals a negative association between group involvement and self-rated happiness. This suggests that individuals living in neighborhoods with a higher number of group memberships were less likely to report increased happiness (OR = 0.861, *p* = 0.096). Our findings thus contradict some previous studies that have demonstrated a positive relationship between greater public participation and elevated happiness [9,10,55]. The discrepancy in these results indicates that the relationship between public participation and happiness is complex and may vary across different contexts, such as during a pandemic compared to a period of normalization.

Furthermore, there is little evidence to suggest that mobility control measures in Chinese neighborhoods have a negative impact on residents’ happiness (OR = 1.187, *p* = 0.414), which is in contrast to findings from studies conducted in Western countries. According to research by [56], there are distinct psychological outcomes associated with voluntary versus involuntary isolation at home. The adverse effects observed were primarily due to restrictions on personal liberty experienced by those who were involuntarily isolated, whereas voluntary isolation was linked to less distress. This perspective highlights the significance of solidarity and cooperation among Chinese citizens in managing the crisis, as the majority of Wuhan residents were voluntarily confined to their homes. These observations underscore the fact that urban governance and happiness outcomes can differ across various countries and localities.

Models 3 and 4 highlight public satisfaction as a crucial social mechanism for explaining how governance measures affect residents’ self-reported happiness. Specifically, Model 4 reveals that governance measures can significantly impact residents’ satisfaction with governance. Factors such as diverse public service delivery, extensive group involvement, and timely response were positively associated with respondents’ governance satisfaction. Additionally, the coefficient of living essentials provision (measured by the material shortage index) was negative and significant (Coef. = −0.073, with the corresponding odds ratio of 0.930, *p* = 0.017). Furthermore, Model 3 indicates a strong relationship between governance satisfaction and self-rated happiness. This suggests that residents were considerably more likely to report higher happiness scores if they were more satisfied with neighborhood governance (OR = 1.228, *p* = 0.002). In summary, neighborhood governance, utilizing the objective dimension (i.e., the concerning measures), can influence residents’ self-rated happiness via the perceptual dimension (i.e., satisfaction), signifying the presence of a social mechanism.

### 4.3. Hukou-Based Social Inequality of Neighborhood Governance

In Table 3, Model 5, we illustrated the extent to which the strength of association between neighborhood governance and self-rated happiness varied by *hukou* type. The coefficient of the interaction term between group involvement and *hukou* type was positive and significant at a 90% significance level. This means that group involvement adversely influenced migrants’ self-rated happiness more strongly than locals (OR = 1.463, *p* = 0.086). Moreover, the positive impact of prompt medical treatment on migrants’ self-rated happiness was also stronger relative to that of locals (OR = 0.693, *p* = 0.042).

Model 6 indicates that there are also differences in the effects of governance measures on public satisfaction between locals and migrants. However, only the interaction term between prompt medical treatment and *hukou* type was found to be significant, implying that prompt medical treatment can boost migrants’ governance satisfaction more than that of locals. Overall, the analyses conducted above suggest that the relationship between neighborhood governance measures, satisfaction, and residents’ self-rated happiness varied between migrants and locals. Improving migrants’ access to medical care was an important aspect of neighborhood governance during the pandemic that significantly contributed to migrants’ happiness.

As for controlled variables (Model 1), only three variables, self-reported physical health, living arrangement, and risk exposure to COVID-19, have a significant effect on residents’ self-rated happiness. People with a better physical health status exhibited higher happiness than those with poorer health. While those residing in neighborhoods with confirmed or suspected cases and with parents had lower happiness than those who did not.

### 4.4. Robustness Tests

Table A1 in Appendix A shows the results of robustness checks as described in Section 3.3. Our analysis using self-rated mental health as the dependent variable (Models 1–3) produced results that were mostly consistent with those obtained when self-rated happiness was employed. Neighborhood governance significantly affects individuals’ self-rated mental health. Public service delivery, prompt medical treatment, and governance satisfaction were significant and positive predictors, while the provision of life’s necessities (measured by the material shortage index) had a negative impact. Moreover, there was a *hukou*-based social inequality in neighborhood governance (Model 4, Table A1 in Appendix A). Overall, the above empirical analyses confirm that our findings are robust.

## 5. Discussion

Numerous scholars have speculated that China’s success can be attributed to urban governance at the ‘base’ level and its efficacy in combating the common threat [14,16,17,57]. Based on first-hand data collected during Wuhan’s first lockdown, we have revisited the city’s neighborhood governance, observed the underlying social inequality issues, and examined their happiness effects. Our findings suggested that, during a crisis, neighborhood governance (i.e., comprising measures and satisfaction) can significantly influence residents’ self-rated happiness, confirming Hypothesis 1 (H1). Building sound social infrastructures, such as providing diversified public services, assuring basic necessities and timely medical care, and so on, is critical to preserving people’s happiness and mitigating the negative impacts of external risks such as COVID-19. Nevertheless, seemingly good governance does not always result in increased happiness. In our particular case, higher public participation had negative consequences. While group involvement has been shown to strengthen individuals’ sense of community and social capital, which can help mitigate the negative effects of disasters and sustain collective happiness [16,58,59], this positive effect is not always present. Research demonstrated that group involvement has a U-shaped curvilinear effect on mental health, indicating that too much group involvement may have diminishing returns [10]. One study further emphasized the social capital’s ‘declining marginal productivity’, highlighting the potentially diminishing advantage that may emerge from increasing group involvement [60]. This may be due to the possibility of multiple factions/memberships engaging in neighborhood governance, each with its own set of expectations. This can lead to power- and resource-related competition, opposition, and conflicts, which may expose citizens to tensions, negative emotions, and poorer mental health statuses. This situation may explain why group participation was found to be negatively associated with people’s happiness in the case of Wuhan.

In the aforementioned relationships, satisfaction with governance has been identified as a significant social mechanism, which supports Hypothesis 2 (H2). In particular, as part of a broader framework of good governance, prompt medical treatment is an important governance measure that can contribute more than any other governance measure to enhance citizen satisfaction and happiness. Not only that, it is indeed an interesting finding that higher group involvement may lead to increased governance satisfaction but decreased happiness. This suggests that group involvement can have complex effects on individuals’ wellbeing, with positive effects on certain aspects of life but negative effects on others. The possibility is that social structural variables, like the *hukou* system, can cause happiness disparities among different social groups. This is an important issue to consider in the context of urban governance and social inequality. Overall, this finding underscores the need for a more nuanced understanding of the relationship between group involvement, governance satisfaction, and happiness, as well as the role of social structural factors in shaping individuals’ happiness.

Our finding also supported Hypothesis 3 (H3), which revealed a substantial difference in the impact of neighborhood governance on self-rated happiness between migrants and locals, exposing the *hukou*-based social inequality of urban governance. It is not surprising that migrants with greater access to medical support report higher governance satisfaction and self-rated happiness. The unequal treatment between migrants and locals can be attributed to a variety of complex factors, with the *hukou* system being one of the root causes [19,20]. In China, the *hukou* system’s unequal “opportunities” such as limited access to public services provided by the community, can result in unequal “benefits” such as disparities in happiness (Figure 1). Migrants on the “dark side” of the benefits derived from the *hukou* system [19] have been disproportionately affected by this epidemic, highlighting the significance of healthcare coverage in the inflow cities for the social resilience of migrants.

Additionally, it is insightful that the adverse effects of group involvement on migrants’ self-rated happiness are stronger than those of locals. In other words, decentralization is not always oriented toward better results since even its negative impact differed among different social groups. Intriguingly, conflicts and struggles resulting from increased public participation would make it harder for migrants to receive necessary public services. Moreover, these harmful consequences might be too intense for them due to their poor active participation and decision-making rights in urban governance, particularly in their neighborhoods. This finding also corroborates that of some researchers who claimed that the efficacy of governance was also related to the engagement mode such as positive and orderly cooperation, which were more conducive to community stability than vast memberships [3,14,16].

These findings do also have important policy implications. First, neighborhood governance can act as a stress reliever, mitigating external risks and ensuring residents’ happiness. To achieve this, community services and management systems should be enhanced and optimized, and social infrastructures should be built to provide diversified services, secure the necessities of life, and offer medical assistance. Second, based on the framework of “people-centered” urbanization, neighborhood governance, on the one hand, should improve satisfaction to enhance society’s wellbeing by designing incentive programs to motivate community workers to provide better and more decent services for residents, particularly in high-risk communities. On the other hand, neighborhood governance should go beyond the *hukou* reform to address the needs and priorities of vulnerable populations to alleviate social inequality across the city. For instance, a spectrum of strategies should be undertaken, including integrating migrants into the healthcare and social service systems of neighborhood planning and reinforcing migrants’ involvement and empowerment in neighborhood management, thereby creating more inclusive communities.

Some of the limitations of this study are noteworthy. First, governments operate within complex spatial and geographical boundaries, driven by the broad constellation of social, political, and economic forces [2]. Hence, the findings of Wuhan’s research results may not apply to other areas in China. Therefore, comparative research should be carried out across other Chinese cities to validate the generalizability of the evidence. Second, our results may inevitably be biased since they are derived from a single self-rated happiness survey, notwithstanding that we have conducted a robustness test. To produce a more accurate inference, future research plans should incorporate supplementary surveys focusing on the multidimensional measurements of happiness in China. Third, we overlooked some contextual factors related to the epidemic that may impact individual risk perceptions and, subsequently, happiness. While we did take into account residents’ exposure to the outbreak by considering the presence of suspected or confirmed cases in the community (as outlined in Section 3.2), our analysis remains insufficient. To comprehensively understand the situation, future studies should also consider other factors such as population density [61], proximity to the epicenter (Huanan seafood market) [62], availability of neighborhood social supports [63], satisfaction with information disclosure [64], etc.

## 6. Conclusions

The COVID-19 pandemic has amplified pre-existing urban governance issues. Since the crux of previous research centers on the reactions of governance across national, local, and neighborhood scales, few studies have examined the social implications of neighborhood governance, particularly on people’s happiness. Our study, based on micro-individual survey data collected during Wuhan’s initial lockdown in 2020, found that neighborhood governance such as diverse public services delivery, essential supplies provision, and prompt medical treatment had a positive effect on residents’ happiness. However, it is worth noting that active governance actions do not always lead to positive outcomes. For example, increased group participation may lead to decreased happiness due to heightened social conflicts. Importantly, the consequences varied between local residents and migrants, highlighting *hukou*-based social inequality. The unexpected outcomes and the identification of social inequality issues within the framework of urban governance contribute to the theoretical advancement of current knowledge, particularly within the local context, with implications for addressing the challenges posed by the pandemic. We contend that the pandemic’s impact on citizen happiness is the result of both the immediate social crisis caused by the pandemic and long-standing structural inequalities. The unequal distribution of public services, healthcare, and others between locals and migrants highlights the disparities in resource access depending on immigrant status. These discrepancies intensify the wellbeing gaps experienced by different groups, indicating a double inequality of ‘opportunities’ and ‘benefits’ in China’s urban governance that is based on the *hukou* system.

## Figures and Tables

**Table 1:** Descriptive statistics of variables (n = 790).

	Mean (SD)
**Self-rated happiness**	3.37 (1.23)
**Individual attributes**	
Age (%)	
36 and below	44.43
Others	55.57
Gender (%)	
Male	48.61
Female	51.39
Education (%)	
Under Junior Secondary Schools	3.92
Senior Secondary Schools and College	31.52
Undergraduate and above	64.56
Self-reported physical health	4.44 (0.78)
Monthly household income (%)	
Low	17.09
Medium	61.52
High	21.39
Living arrangement (with parents) (%)	
No	56.08
Yes	43.92
*Hukou* type (%)	
Migrants	18.73
Locals	81.27
Risk exposure to COVID-19 ^1^ (%)	
Low	22.91
High	77.09
**Neighborhood governance**	
Objective dimension	
Public service delivery	2.26 (1.38)
Mobility control (%)	
No	12.66
Yes	87.34
Group involvement	1.72 (0.84)
Provision of life’s necessities ^2^	3.56 (2.27)
Prompt medical treatment	3.07 (1.08)
Perceptional dimension	
Governance satisfaction	2.94 (1.26)

^1^ Suspected or confirmed cases in communities (Yes: High risk; No: Low risk). ^2^ A higher index of material shortage suggests a lower provision of life’s necessities for neighborhoods.

**Table 2:** Ordinal Logit Model results of the impacts of neighborhood governance on self-rated happiness ^1^.

	Model 1 (DV: Self-Rated Happiness)		Model 2 (DV: Self-Rated Happiness)		Model 3 (DV: Self-Rated Happiness)		Model 4 (DV: Governance Satisfaction)	
	OR	*SE*	OR	*SE*	OR	*SE*	OR	*SE*
Age (*ref*: 36 and below)	0.124	−0.700	0.989	0.138	1.038	0.146	0.601 ***	0.085
Gender (*ref*: Male)	0.927	0.123	0.900	0.122	0.911	0.123	0.887	0.120
Self-reported physical health	2.411 ***	0.213	1.838 ***	0.169	1.825 ***	0.169	1.071	0.099
Monthly household income (*ref*: Low)								
Medium	1.284	0.233	1.224	0.226	1.223	0.226	0.970	0.184
High	0.804	0.174	0.841	0.188	0.883	0.199	0.631 **	0.143
Living with parents (*ref*: No)	0.645 ***	0.090	0.735 **	0.104	0.746 **	0.106	0.833	0.117
Risk exposure to COVID-19 (*ref*: Low)	0.587 ***	0.093	0.733 *	0.121	0.803	0.135	0.456 ***	0.077
**Neighborhood governance**								
Objective dimension								
Public service delivery			1.149 **	0.067	1.100	0.066	1.499 ***	0.088
Mobility control (*ref*: No)			1.187	0.248	1.104	0.233	1.840 ***	0.403
Group involvement			0.861 *	0.077	0.847 *	0.076	1.203 **	0.110
Provision of life’s necessities			0.895 ***	0.027	0.899 ***	0.027	0.930 **	0.028
Prompt medical treatment			2.007 ***	0.142	1.849 ***	0.139	2.272 ***	0.173
Perceptional dimension								
Governance satisfaction					1.228 ***	0.080		
Log-likelihood	−1127.481		−1061.309		−1056.361		−1048.765	
Pseudo R^2^	0.061		0.116		0.120		0.143	
N	790		790		790		790	

^1^ * *p* < 0.1; ** *p* < 0.05; *** *p* < 0.01.

**Table 3:** Associations between neighborhood governance measures, satisfaction, and self-rated happiness depend on the *hukou* type.

	Model 5 (DV: Self-Rated Happiness)		Model 6 (DV: Governance Satisfaction)	
	OR	*SE*	OR	*SE*
Age (*ref*: 36 and below)	1.062	0.157	0.630 ***	0.093
Gender (*ref*: Male)	0.913	0.124	0.887	0.120
Self-reported physical health	1.838 ***	0.171	1.071	0.099
Monthly household income (*ref*: Low)				
Medium	1.179	0.221	0.964	0.185
High	0.852	0.194	0.614 **	0.141
Living with parents (*ref*: No)	0.764 *	0.110	0.864	0.124
Risk exposure to COVID-19 (*ref*: Low)	0.812	0.142	0.472 ***	0.083
Hukou type (*ref*: Migrants)	1.316	0.982	2.060	1.626
**Neighborhood governance**				
Objective dimension				
Public services delivery	1.269 *	0.173	1.456 ***	0.194
× *Hukou*	0.844	0.127	1.052	0.155
Mobility control (*ref*: No)	0.706	0.326	1.484	0.697
× *Hukou*	1.745	0.910	1.294	0.687
Group involvement	0.621 **	0.123	1.131	0.234
× *Hukou*	1.463 *	0.325	1.060	0.245
Provision of life’s necessities	0.929	0.068	0.926	0.069
× *Hukou*	0.961	0.077	1.000	0.081
Prompt medical treatment	2.501 ***	0.411	3.092 ***	0.473
× *Hukou*	0.693 **	0.125	0.675 **	0.112
Perceptional dimension				
Governance satisfaction	1.158	0.179		
× *Hukou*	1.062	0.179		
Log-likelihood	−1051.785		−1045.464	
Pseudo R^2^	0.124		0.146	
N	790		790	

* *p* < 0.1; ** *p* < 0.05; *** *p* < 0.01.

## Data Availability

The data of this study are available from the corresponding author upon reasonable request.

## Appendix A. Results of Robustness Tests

Table A1 in Appendix A shows the results of robustness checks, as described in Section 3.3, with self-rated mental health as the dependent variable (Models 1–4).

**Table A1:** Results of robustness tests.

DV: Self-Rated Mental Health	Model 1		Model 2		Model 3		Model 4	
	OR	*SE*	OR	*SE*	OR	*SE*	OR	*SE*
Age (*ref*: 36 and below)	0.915	0.139	0.930	0.144	0.978	0.152	0.929	0.151
Gender (*ref*: Male)	0.547 ***	0.080	0.518 ***	0.077	0.521 ***	0.078	0.497 ***	0.076
Self-reported physical health	6.148 ***	0.674	5.300 ***	0.596	5.324 ***	0.602	5.491 ***	0.628
Monthly household income (*ref*: Low)								
Medium	1.374	0.269	1.382	0.275	1.384	0.277	1.439 *	0.292
High	0.912	0.213	0.982	0.236	1.046	0.253	1.132	0.280
Living arrangement (*ref*: No)	0.542 ***	0.083	0.570 ***	0.089	0.581 ***	0.091	0.582 ***	0.092
Risk exposure to COVID-19 (*ref*: Low)	0.584 ***	0.105	0.663 **	0.124	0.731 *	0.139	0.681 *	0.135
*Hukou* type (*ref*: Migrants)							2.971	2.670
**Neighborhood governance**								
Objective dimension								
Public services delivery			1.116 *	0.070	1.064	0.069	1.337 *	0.204
× *Hukou*							0.754 *	0.127
Mobility control (*ref*: No)			1.119	0.257	1.029	0.239	0.270 **	0.155
× *Hukou*							4.907 **	3.093
Group involvement			0.878	0.090	0.859	0.088	0.964	0.227
× *Hukou*							0.866	0.227
Provision of life’s necessities			0.947 *	0.031	0.952	0.032	1.016	0.085
× *Hukou*							0.932	0.084
Prompt medical treatment			1.526 ***	0.117	1.409 ***	0.114	2.171 ***	0.438
× *Hukou*							0.618 **	0.135
Perceptional dimension								
Governance satisfaction					1.243 ***	0.089	1.093	0.191
× *Hukou*							1.172	0.222
Log-likelihood	−803.246		−782.740		−778.101		−769.893	
Pseudo R^2^	0.203		0.223		0.228		0.236	
N	790		790		790		790	

* *p* < 0.1; ** *p* < 0.05; *** *p* < 0.01.
